# Microglial polarization in TBI: Signaling pathways and influencing pharmaceuticals

**DOI:** 10.3389/fnagi.2022.901117

**Published:** 2022-08-01

**Authors:** Yun-Fei Li, Xu Ren, Liang Zhang, Yu-Hai Wang, Tao Chen

**Affiliations:** Department of Neurosurgery, The 904th Hospital of PLA, Medical School of Anhui Medical University, Wuxi, China

**Keywords:** TBI, microglial polarization, cytokine, signaling pathway, inhibitors/agonists

## Abstract

Traumatic brain injury (TBI) is a serious disease that threatens life and health of people. It poses a great economic burden on the healthcare system. Thus, seeking effective therapy to cure a patient with TBI is a matter of great urgency. Microglia are macrophages in the central nervous system (CNS) and play an important role in neuroinflammation. When TBI occurs, the human body environment changes dramatically and microglia polarize to one of two different phenotypes: M1 and M2. M1 microglia play a role in promoting the development of inflammation, while M2 microglia play a role in inhibiting inflammation. How to regulate the polarization direction of microglia is of great significance for the treatment of patients with TBI. The polarization of microglia involves many cellular signal transduction pathways, such as the TLR-4/NF-κB, JAK/STAT, HMGB1, MAPK, and PPAR-γ pathways. These provide a theoretical basis for us to seek therapeutic drugs for the patient with TBI. There are several drugs that target these pathways, including fingolimod, minocycline, Tak-242 and erythropoietin (EPO), and CSF-1. In this study, we will review signaling pathways involved in microglial polarization and medications that influence this process.

## Introduction

Traumatic brain injury (TBI) occurs when the brain is hit by an external force, with a series of serious consequences. The global annual incidence of TBI exceeds 50 million individuals, and a study has said that half of the world’s population is likely to have one or more TBIs in their lifetime (Maas et al., 2017). The morbidity of TBI in China is estimated to be approximately 0.013%, which is similar to the rates reported in other countries (Jiang et al., 2019). China has the world’s largest population, which means that China would also have the most individuals who would encounter a TBI, making TBI a major public health concern in China. In another review, China was found to have approximately 770,060–890,990 new cases of TBI every year, and the average cost of patients ranges from ¥28,000 to 129,000 (Gao et al., 2020). Damage to neuronal tissues following TBI has two stages: the primary injury, which is directly caused by an external force when TBI occurs; and the secondary injury, which follows primary insult and causes tissue and cellular damage. The primary injury in TBI is largely irreversible, so the research focuses on changing the course of secondary injury. However, the pathophysiological mechanisms of the secondary injury are not well understood, including but not limited to excitotoxicity, mitochondrial dysfunction, oxidative stress, lipid peroxidation, neuroinflammation, axon degeneration, and apoptotic cell death (Ray et al., 2002). Neuroinflammation is the most intensively studied of these pathological mechanisms. In accordance with Bergold (2016) review, neuroinflammation plays a major role in traumatic brain damage, regulating the inflammatory process that can effectively treat TBI.

Microglia are cells present in the adult mammalian brain that account for 5–20% of all glial cells. They are derived from erythroid myeloid precursors in the embryonic yolk sac and are distributed within the embryonic mouse brain (Ginhoux et al., 2010). Microglia are vital to CNS homeostasis and are involved in the evolution of a variety of neurological pathophysiological states such as neuropsychiatric disorders, neurodegeneration, neuroinflammation, sterile injury responses, and infectious diseases (Nayak et al., 2014). The most important role of microglia is in neuroinflammation. Under normal conditions, microglia assume a neural-specific phenotype (Schmid et al., 2009) and retain a relative quiescent surveillance phenotype for constant monitoring of the brain parenchyma (Davalos et al., 2005). Modulation of neuroinflammation following TBI may require addressing both inflammatory pathways and facilitating repair (Corrigan et al., 2016). Within injured tissues, microglia exist in various states of activation and retain the capability to shift their functional phenotype during the inflammatory response (Stout et al., 2005). When an injury occurs, microglia activation can be divided into two processes. First, microglia polarize toward the pro-inflammatory (M1) phenotype that produces pro-inflammatory cytokines, such as TNF-α, interleukin (IL-1β, IL-12), present antigens, and express high levels of inducible NO synthase (iNOS) for NO production (Gordon and Taylor, 2005; Villalta et al., 2009). Then, microglia polarize to the M2 phenotype, expressing anti-inflammatory cytokines (IL-4, IL-10, IL-13, and TGF-β), arginase-1 (Arg-1), CD206, and chitinase-3-like-3 (Ym1 in rodents) (Colton, 2009; Henkel et al., 2009).

Classical activation (M1) is defined as the stimulation of microglia by external factors or elements, such as microorganisms and some cytokines, resulting in high expression of pro-inflammatory cytokines and an enhanced capacity for phagocytosis. Activating substances include two aspects, external matters, such as exogenous particles and bacteria, and endogenous matters, such as interferon-γ, TNF, and cell debris. The M1 phenotype is usually associated with the host defense against intracellular pathogens (Mackaness, 1977; Gordon and Taylor, 2005; Dale et al., 2008). The M2 phenotype is more complex (see Table 1). The M2 phenotype can be divided into three subtypes: M2a, M2b, and M2c. In the early 1990s, the concept of macrophage alternative activation was developed largely based on research showing a role for IL-4 in the induction of an alternative (M2) activation state (Stein et al., 1992) inducing the expression of anti-inflammatory cytokines IL-4, IL-10, IL-13, and TGF-β and arginase-1 (Arg-1), CD206, and chitinase-3-like-3 (Ym1 in rodents) (Colton, 2009; Henkel et al., 2009). M2 microglia play an important role in allergy response, parasite clearance, inflammatory dampening, tissue remodeling, angiogenesis, immunoregulation, and tumor promotion (Sica and Mantovani, 2012). Further studies have found that the M2a activation state is induced by parasitic products or associated signals (IL-4 and IL-13) with a longer term function for resolution and repair (Rutschman et al., 2001; Gordon and Taylor, 2005; Lawrence and Natoli, 2011; Wynn et al., 2013). In this case, the IL-4 receptor and its downstream molecules lead to the inhibition of nuclear factor kappa B (NF-κB) signaling and subsequent inhibition of M1 phenotype activation. M2b polarization is observed with the triggering of Fc-γ receptors, TLRs, and immune complexes (Murray et al., 2014). M2c polarization occurs in response to specific anti-inflammatory factors such as IL-10, TGF-β, and glucocorticoids (Vodovotz et al., 1993; Gordon and Taylor, 2005; Martinez et al., 2008). The alternative activation of microglia has been proposed for 40 years, but there is crosstalk among the subtypes of M2 phenotype, indicating that the polarization subtypes and functions of M2 microglia subtypes have not been fully understood so far. A recent study showed that energy metabolism plays an important role in the process of microglial polarization (Ghosh et al., 2018). Energy metabolism in microglia is mainly supplied by the tricarboxylic acid cycle (TCA). When microglia are induced to move toward the M1 phenotype by the external microenvironment, they will inhibit mitochondrial oxidative phosphorylation (OXPHOS) (Baseler et al., 2016). In contrast, “alternatively activated” microglia adopt a metabolic program dominated by fatty acid-fueled OXPHOS. More studies focus on how to facilitate the two processes to regulate microglial polarization. Compared with serum-cultured microglia, microglia cultured in a defined medium performed less well after the same stimulation, which was related to their metabolic state (Montilla et al., 2020). However, how metabolic states affect microglial polarization levels by regulating related cellular signaling pathways has not been clearly explained. Some researchers have focused on the mechanistic target of the rapamycin (mTOR) pathway. mTOR inhibition led to decreased production of LPS-induced pro-inflammatory cytokines by suppressing glycolysis (Hu et al., 2020). mTOR receptor inhibits the classical polarization of M1 microglia by enhancing anaerobic glycolysis in microglia and weakening mitochondrial function. Now, more and more studies have shown that microglia are not simply divided into M1 and M2 types, but also include other types, such as rod microglia. In addition to the classic M1/M2 classification of microglia, rod-shaped microglia, which were first introduced in 1899 (Au and Ma, 2017), are the current focus of research. Despite 100 years of study, little is known about the specific functions of rod-shaped microglia due to the lack of technology to cultivate rod-shaped microglia in vitro. Rod microglia, also called bipolar microglia, were observed in a single trajectory with their highly polarized processes seemingly connected (Ziebell et al., 2012). In a recent study, rod microglia were identified as an abundance of iba1-positive microglia with phagocytosis. With aging society as an aggravating factor, individuals with AD place considerable pressure on the healthcare systems in society. Due to its contribution to the development of AD, rod microglia are now the focus of several studies (Bachstetter et al., 2015). A cost-effective and highly reproducible method has been established to enrich bipolar/rod-shaped microglia in vitro (Tam and Ma, 2014; Tam et al., 2016), leading to accelerated research on these cells over the past few years. In the optic nerve transection (ONT) model, the appearance of rod microglia is not only closely related to injury time but is also related to the cortical position (Yuan et al., 2015). The distribution density of rod-shaped microglia differs in different cortices, which may be related to the different phenotypes of static microglia induced by specific microenvironment in different parts of the brain after injury. The process by primitive microglia are transformed into rod microglia is mediated by the granulocyte-macrophage colony stimulating factor (GM-CSF) and intracellular calcium concentration ([Ca^2+^]_*i*_) of microglial cells (Suzumura et al., 1990; Frei et al., 1992). In the ONT model, rod microglia showed strong phagocytosis, and rod-shaped microglia disappeared after tissue fragments were removed (Yuan et al., 2015). Therefore, cell debris may also be factors inducing the formation of rod microglia. The development of new therapeutic interventions by switching the microglial phenotype from amoeboid to rod microglia might shed new light on pathogenesis and identify targets for treating neurodegenerative diseases. As mentioned above, in addition to the classical and alternative pathways, there are other pathways involved in microglia activation. Therefore, the current international description of the polarization state of microglia focuses more on its function, following which they are classified into “pro-inflammatory” (or the M1) phenotype and the “anti-inflammatory” (or the M2) phenotype. Different microglial subtypes are dominant between 1 and 3 weeks after injury (Jin et al., 2012). Indeed, the M2 phenotype peaks at 1 week of TBI, decreases thereafter, and returns to normal level within 4 weeks. However, the M1 phenotype increases 4 weeks after injury. Different results were reported by Kumar et al. (2016) who found that M1 and M2 phenotypes were activated after TBI, but the M2 phenotype was replaced by the M1 phenotype at 7 day post-injury. This shift toward the M1 phenotype was associated with increased neurodegeneration. The focus of this study is to identify the process so that the transformation could be prevented or slowed down, as well as to prolong the expression of M2 microglia. Here, we organized a number of cellular signaling pathways that influence the transformation of microglial phenotypes.

**TABLE 1:** The characteristics of the different sub-types of microglia.

Microglia phenotype	Activation substances	Cell surface protein	Secrete protein	Function
M1	LPS, Interferon-γ, TNF	CD16, CD32 CD86, MHC-II	IL-1β	Pro-inflammatory, Boost inflammation Todd et al., 2019
			IL-6	Pro-inflammatory, Boost inflammation Trapero and Cauli, 2014
			IL-12 /p70	Pro-inflammatory, Boost inflammation Sun et al., 2015
			TNF	debris removal Probert, 2015
			IFN-γ	positive feedback Orihuela et al., 2016
			CCL5	Pro-inflammatory, Boost inflammation Skuljec et al., 2011
			CXCL1	Sterilization Stamatovic et al., 2020
			CXCL10	apoptotic cell removal Tassiulas et al., 2007
M2a	IL-4, IL-13, TREM2	CD206	IL-1Ra	Wound healing Martinez et al., 2008
			IL-4	Anti-inflammatory, increases microglia and macrophage phagocytosis Zhao et al., 2015
			TGFβ	Anti-inflammatory Zhong et al., 2018
M2b	TLRs + immune complexes	IL-6, VEGF, IGF-1, CD86, TNF-α, CD64	Ym1	Immunoregulatory Adams, 1989; Martinez et al., 2008
			Arg1	Suppresses inflammation Munder et al., 2005
			IL-10	Anti-inflammatory, mediate microglia and macrophage phagocytosis Durafourt et al., 2012
			IL-4Rα	tissue stabilization Mozo et al., 1998
			G-CSF	Mediates microglia and macrophage survival, proliferation and differentiation Roberts, 2005
			FIZZ1	Anti-inflammatory; induction depends on IL-4 and IL-13 Raes et al., 2002
M2c	IL-10	CD163 CD206,	TGF-B	Immunosuppressive Martinez et al., 2008; Bogdan et al., 1992
			SLAM	immune regulation Dragovich and Mor, 2018
			Sphk-1	tissue repair Yu et al., 2009a
			THBS1	ECM synthesis Yamashiro et al., 2020
			HMOX-1	reduce oxidative stress Zhu et al., 2017

## Cell signaling pathways that influence microglial polarization

Microglia play a very important role in neuroinflammation, and many signaling pathways are involved in their polarization. Clarifying the interaction between these pathways and their influence on microglia is very important for regulating the polarization of microglia, while also providing a basis for the development of therapeutical drugs. Here, we summarize some of the classical and important signaling pathways involved in microglial polarization.

### Toll-like receptor-4/NF-κB pathway

A toll-like receptor (TLR) is a pattern-recognition receptor that detects microbial components, and the TLR family consists of 13 members (Takeda and Akira, 2015). TLR-4 is expressed in microglia, astrocytes, and macrophages in the brain, and can be activated by LPS (Badshah et al., 2016). NF-κB is a key regulator of immune development, immune responses, and inflammation (Mitchell et al., 2016). The canonical pathway of the NF-κB pathway is triggered by TLR, and RelA is an activating substance between them. The pathway function is to regulate the expression of pro-inflammatory and cell survival genes (Karin et al., 2006; Lawrence, 2009).

In microglia, TLR-4/NF-κB is a traditional transcription factor that is activated by lipopolysaccharide (LPS) and regulates the expression of most M1-signature genes encoding pro-inflammatory cytokines (Taetzsch et al., 2015). Polarization of microglia in the direction of the M1 phenotype induced by the TLR-4 signaling pathway leads to damage to white matter tracts in the corpus callosum (Yang et al., 2018). In another study, the development of PD-associated neuroinflammation was reduced in TLR-4-deficient Parkinson’s disease (PD) mouse models compared with normal mice without PD (Campolo et al., 2019). TLR-4 could be considered an encouraging therapeutic target in neurodegenerative disease.

The diagnosis and treatment of patients with TBI now focus on inhibiting the TLR-4/NF-κB pathway. For instance, the viral inhibitory peptide of TLR-4 (VIPER) interacts with the myeloid differentiation factor 88 (MyD88) adaptor-like (Mal) and TRIF-related adaptor molecule (TRAM) to inhibit the TLR-4/NF-κB pathway and attenuate microglia activation (Lysakova-Devine et al., 2010; Masson et al., 2015). Vascular endothelial growth inhibitor (VEGI) could alleviate the post-traumatic excessive inflammatory response and remit the secondary brain damage by downregulating the expression of the TLR-4/NF-κB signaling pathway and inflammatory cytokines (Gao et al., 2015). Therefore, the TLR-4/NF-κB pathway can be said to promote M1-type polarization after TBI.

### Janus kinase/signal transducers and activators of transcription pathway

The Janus Kinase/Signal Transducers and Activators of Transcription (JAK/STAT) signaling pathways have been recognized as one of the most important pathways in mediating innate and adaptive immunity (O’Shea and Plenge, 2012). The JAK family has four main members with over 1,000 amino acids each, and their molecular weights range from 120 to 140 kDa (Cai et al., 2015). The STAT family in the cytoplasm is a downstream target of JAKs, which consists of seven members with molecular weights ranging from 79 to 113 kDa (Darnell, 1997; Boengler et al., 2008; Yu et al., 2009b). When JAK is attached to the ligand, STAT is phosphorylated, dimerized, and transported into the nucleus to regulate the expression of related genes. The JAK/STAT pathway is well known to modulate various signals to maintain homeostasis in inflammatory conditions. It induces neuroinflammatory diseases such as PD and multiple sclerosis (MS) by modulating microglial polarization (Xin et al., 2020). The phenotype of microglia changes to M2 from M1 after the JAK/STAT signaling pathway phosphorylation process is inhibited, and the microglia then produce fewer inflammatory cytokines (Qu et al., 2019).

CNS homeostasis is disrupted for which microglia are overactivated, leading to an inflammatory storm after TBI. The severity of this inflammatory storm can be regulated via the JAK/STAT pathway to improve the prognosis of TBI. JAK2/STAT1 is a pro-apoptotic pathway that upregulates the expression of Fas, FasL, and IRF-1 and inhibits the anti-apoptotic NF-κB (Chin et al., 1997; Kumar et al., 1997; Wang et al., 2000). Additionally, this pathway promotes the polarization of microglia into the M1 phenotype (Porro et al., 2019). JAK2/STAT1 activation induces the expression of genes encoding IL-1β, TNF, and CXC motif chemokine 10 (CXCL10), indicating that the JAK2/STAT1 pathway promotes the polarization of macrophages into the M1 phenotype (Lawrence and Natoli, 2011). When the JAK2/STAT3 pathway was activated by paraquat, microglia were found to polarize into the M1 phenotype, consequently causing inflammatory damage to the hippocampus (Fan et al., 2022). Unlike this, the JAK2/STAT6 pathway promotes polarization of microglia to the M2 phenotype (Yang et al., 2017). The effect of the JAK/STAT family on microglia is not fully understood; thus, further studies are needed to clarify the different JAK/STAT pathways that could influence the polarization of microglia and the therapeutic needs of TBI.

### HMGB1

High mobility group box 1 (HMGB1) is a non-histone nuclear protein with high electrophoretic mobility of 215 amino acids. HMGB1 was first described by Goodwin and Johns (1977). It is estimated that each nucleus contains approximately 1 × 10^6^ HMGB1 molecules, which is only just lower than the core histone (Romani et al., 1979). The function of HMGB1 in the nucleus is DNA-binding activity, DNA chaperone, and DNA-bending activity (Kang et al., 2014). Extracellular HMGB1 functions as an immune adjuvant to trigger a robust response to activation or suppression of cells including T cells, macrophagocytes, and dendritic cells.

HMGB1 plays an important role in regulating the systemic inflammatory response in infectious diseases, and the serum level of HMGB1 in patients with sepsis is elevated (Wang et al., 1999). Current studies show that the regulation of HMGB1 on local inflammation can effectively change the occurrence, development, and prognosis of diseases such as stroke, TBI, PD, epilepsy, and Alzheimer’s disease (AD) via the regulation of microglial polarization (Nishibori et al., 2019). Paudel et al’s paper discussed the contribution of the HMGB1/TLR4/RAGE signaling pathway in TBI and other neuroinflammatory diseases, arguing the possibilities of HMGB1 as a common viable biomarker of TBI (Paudel et al., 2018).

A previous study showed that early treatment with anti-HMGB1 monoclonal antibody (m-Ab) might be a promising strategy for TBI (Okuma et al., 2012). One in vitro study demonstrated that HMGB1 induced the polarization of microglia toward the pro-inflammatory phenotype (Fan et al., 2020b). The study also demonstrated that inhibiting the HMGB1-RAGE axis prevented pro-inflammatory microglial polarization and afforded neuroprotection after SCI in rats. Li et al. (2020) found that the HMGB1 inhibitor BAI could improve acute neurocognitive impairment by HMGB1-mediated inhibition of neuroinflammation in LPS-induced mice.

### Members of the mitogen-activated protein kinases signaling pathway

Members of the mitogen-activated protein kinases (MAPKs) family are typically activated by various mitogenic agents such as growth factors and hormones. This family plays an important role in regulating cell division and differentiation (Gustin et al., 1998). The MAPK family, including extracellular signal-regulated kinase (ERK), p38, and c-Jun *N*-terminal kinase, is a group of signaling molecules that plays an important role in the expression of pro-inflammatory cytokines (Liu et al., 2020). NF-κB is a downstream molecule of MAPK. MAPK/NF-κB pathway plays an important role in regulating the release of inflammatory mediators (Deng et al., 2018).

The MAPK/NF-κB pathway has been shown to be involved in the production of pro-inflammatory mediators in LPS-treated BV2 cells (Do et al., 2020). In spinal cord injury, when this pathway is activated, it induces the production of pro-inflammatory cytokines, including IL-6, TNF-α, or IL-1β, from microglial cells, which is indicative of microglial polarization toward the M1 phenotype (Liu et al., 2020). Methionine sulfoxide reductase A (MsrA) is an enzyme that plays a role in demyelination and has been shown to suppress inflammatory activation of microglia and oxidative stress *via* inhibition of the MAPK/NF-κB signaling pathway (Fan et al., 2020a).

In the CCI mice model, bazedoxifene was found to protect cerebral cognitive functions after TBI and attenuate impairments in blood-brain barrier (BBB) damage by blocking the MAPK/NF-κB signaling pathway (Lan et al., 2019). Research tends to focus almost entirely on using drugs to suppress one specific inflammatory signaling pathway, but one interesting phenomenon is that splenectomy in TBI mice can downregulate the MAPK/NF-κB signaling pathway, thereby inhibiting the polarization of microglia to the M1 phenotype (Chu et al., 2013). Together, these results support a potential role for the MAPK/NF-κB signaling pathway in the modulation of microglial polarization after TBI.

### Peroxisome proliferation-activated receptors-gamma pathway

Peroxisome proliferation-activated receptors (PPARs) are nuclear hormone receptors that directly bind and respond to ligands such as steroids, thyroid hormone, retinoids, cholesterol by-products, and lipids (Chandra et al., 2008). PPARs are composed of three isoforms: PPAR-α, PPAR-β/δ, and PPAR-γ (Berger and Moller, 2002). These receptors contain poorly conserved A/B regions that, in some cases, act as potent transcriptional activators, provide sites of protein phosphorylation, or form direct interactions with other receptor domains or regulatory proteins (Bain et al., 2007). These three isotypes differ from each other in terms of their tissue distribution, ligand specificities, and physiological roles. PPAR-γ is highly expressed in white and brown adipose tissue, and it plays a key role in the regulation of adipogenesis, energy balance, and lipid biosynthesis (Lehrke and Lazar, 2005; Medina-Gomez et al., 2007).

It was previously thought that this receptor only participates in lipoprotein metabolism; however, a recent study showed that PPAR-γ also participates in the regulation of microglial polarization (Zhou et al., 2020). Why does a receptor that is primarily found in adipose tissue cells exert a regulatory effect on microglia? in an article by Fujisaka et al., The M1-to-M2 ratio was increased by a high-fat-diet and decreased by subsequent pioglitazone, PPAR-γ agonist, treatment (Fujisaka et al., 2009). This indicates that lipid metabolism can affect microglial polarization, but its internal molecular mechanisms need more research. It is confirmed that PPAR-γ agonists can be a promising therapy for PD because it suppresses the M1 phenotype and production of pro-inflammatory cytokines (Carta and Pisanu, 2013).

A research team at Zhejiang University, China, was among those who studied the role of PPAR-γ in TBI. They confirmed that axonal injury after TBI can be alleviated by PPAR-γ agonists, which induced microglia polarized to the M2 phenotype (Wen et al., 2018). In another study using a mouse model, Jiang et al., found that the Chinese herb phyllyrin inhibits inflammation of microglia via the PPAR-γ signaling pathway, protecting mice from TBI (Jiang et al., 2020). As discussed above, microglia are polarized to the M1 phenotype when anaerobic glycolysis increases within the cell; thus, the inhibition of microglia via the PPAR-γ signaling pathway may be related to the increase in aerobic glucose metabolism. Further investigations are needed to determine the validity of the PPAR-γ pathway in the polarization of microglia.

## Specific inhibitors/agonists targeting specific signaling

Currently, several promising anti-TBI drugs are undergoing clinical trials (Table 2). Most exert neuroprotective effects by inhibiting M1 phenotype microglia or enhancing M2 phenotype microglia. In this review, we emphasized the importance of the M2 phenotype in microglial responses following TBI, and treatment strategies with a focus on modulating or enhancing microglia with the M2 phenotype.

**TABLE 2:** Anti-TBI drugs.

Drugs	Mode of action	Effects on microglia	Clinical Trial
fingolimod	S1PR activator Zhang and Wang, 2020	Inhibit M1 phenotype polarization	Phase III
minocycline	Inhibiting MAPK-NF-κB signal pathway Yoshida et al., 2020	Inhibit M1 phenotype polarization	Phase II
Tak-242	TLR-4 inhibitor Samarpita et al., 2020	Inhibit M1 phenotype polarization	Phase II
EPO	Anti-inflammatory Zhou et al., 2017	Shift M1 phenotype to M2 phenotype	Phase II
CSF-1	CSF-1R activator Stanley et al., 1978	Shift M1 phenotype to M2 phenotype	Phase II

### Fingolimod

Fingolimod (FTY720, R,2-amino-2[2-(4-octylphenyl) ethyl]-1,3-propanediol)), sold under the brand name Gilenya, was originally synthesized by the Japanese chemist Tetsuro Fujita (Huwiler and Zangemeister-Wittke, 2018). It is a high-affinity agonist of sphingosine-1-phosphate (SP1) receptor (Fujita et al., 1996). SP1 is well known to be involved in immune regulation in the body (Obinata and Hla, 2019). Therefore, FTY720 is involved in immunoregulation.

When FTY720 was synthesized, it was first applied to solid organ transplantation for its function as an agonist of SP1, which regulates inflammatory processes (Napoli, 2000). A growing body of evidence suggests that FTY720 is neuroprotective in CNS injuries due to its effects on the improvement of cognitive function, protection of BBB function, inhibition of apoptosis and inflammation, suppression of oxidative stress, and regulation of autophagy [87]. FTY720 has been used in various models of stroke and neurological disorders (Carreras et al., 2019; Wang et al., 2020; Rajabi et al., 2021).

Fingolimod is the first FDA-approved drug for the treatment of multiple sclerosis (MS) (Brunkhorst et al., 2014). Now, more and more scientists focus on its role in TBI. Consecutive administration of FTY720 for 3 days in a CCI mouse model was found to improve neurological functions and modulate multiple immune responses by attenuating the generally activated microglia and augmenting the M2/M1 ratio accompanied by decreased axonal damage (Gao et al., 2017). On the contrary, in a C57BL/6 mouse model of focal cortical cryo-lesion injury, FTY720 attenuated neuroinflammation but did not alter the lesion size or affect functional recovery (Mencl et al., 2014). These contradictory results may be because of the different types of brain injury in the above studies. There are also some contradictory results regarding the signaling pathway involved in FTY720. FTY720 has been found to significantly transform pro-inflammatory microglia into anti-inflammatory microglia by suppressing autophagy via STAT1 (Hu et al., 2021). Qin et al. found that FTY720 facilitated M1 to M2 switch of microglia via the STAT3 pathway (Qin et al., 2017). FTY720 does mitigate post-traumatic neuroinflammatory responses *via* a variety of signaling pathways.

### Minocycline

Minocycline was first introduced in 1967 as a second-generation tetracycline derivative (Jonas and Cunha, 1982). It has a wide spectrum of activity against both gram-positive and gram-negative bacteria (Garrido-Mesa et al., 2013). Minocycline shares the basic four-ring structure of the other commonly used tetracyclines, having as its chemical characteristic the substitution of a dimethylamino group in the seventh position (Allen, 1976). It is usually prepared as dihydrated hydrochloride and, in that form, has a molecular weight of 530.

Obviously, minocycline is a broad-spectrum antibiotic, but how does it play an anti-inflammatory role? Some proposed mechanisms for its anti-inflammatory properties include the inhibitory effects on the activities of key enzymes, like iNOS and MMPs (Garrido-Mesa et al., 2013). In particular, minocycline has been shown capable of inhibiting the M1 polarization state of microglia in the CNS via inhibition of NF-κB and via interference of the MAPK pathways (Kobayashi et al., 2013). Minocycline also promotes M2 microglial polarization via upregulating the TrkB/BDNF pathway after intracerebral hemorrhage (ICH)(Miao et al., 2018). In addition, the minocycline could shift the activated M1 microglia phenotype into the M2 phenotype.

Minocycline significantly reduced impairments of spatial learning and memory in the water maze test after TBI in mouse models (Lam et al., 2013). The effects of minocycline treatment in an animal model of TBI revealed that the protective effects could be detected in a short term after injury (3 days after injury) but not in long-term therapy (7 days after injury) (Hanlon et al., 2016). Further experiments confirmed that minocycline is ineffective in reducing microglial activation and ameliorating injury-induced deficits following repetitive neonatal traumatic brain injury. However, in mild blast-induced TBI (mb-TBI) models, acute minocycline treatment appears to prevent the development of neurobehavioral abnormalities likely through normalizing damage markers like NSE, NF-H, Tau, S100β, and glial markers of the injury (Kovesdi et al., 2012). In clinical trials, minocycline is beneficial in patients with moderate-to-severe TBI, but the therapeutic effect did not increase with the dose of minocycline administered (Meythaler et al., 2019). As it is an old pharmaceutical agent, further research on the newer applications, time and method of administration of minocycline is required.

### TAK-242

TAK-242 (ethyl (6R)-6-[N-(2-chloro-4-fluorophenyl) sulfamoyl] cyclohex-1-ene-1-carboxylate) is a small molecule that selectively binds to TLR-4, thereby inhibiting TLR-4 signal transduction (Sha et al., 2007). TAK-242 binds selectively to Cys747, which is the intracellular domain of TLR-4 (Matsunaga et al., 2011). It is well known that TLR-4 plays an important role in inflammation, so its antagonist TAK-242 is first applied in anti-inflammation. TAK-242 has a lower molecular weight and liposoluble capacity, which allows it to cross the BBB (Hua et al., 2015).

TAK-242 has become a focus drug for neuroinflammatory disease due to its special molecular structure and anti-inflammatory effects. In AD mice models, TAK-242 administration significantly improved neurological function and increased the expression levels of the M2 phenotype of microglia. The mechanism of the phenomenon may be that TAK-242 modulates MyD88/NF-κB/NLRP3, which is the downstream signaling pathway of TLR-4 (Cui et al., 2020). Neuroinflammation contributes to the progression of amyotrophic lateral sclerosis (ALS). The microglial reaction is attenuated in TAK-242-treated mice (Fellner et al., 2017). Further research showed that TAK-242 also reduces spinal motor neuron loss in ALS; however, this effect did not result in an increased survival rate.

There is evidence indicating that TAK-242 is beneficial to TBI. Pre-injury treatment of mice with TAK-242 significantly enhances cognitive functional recovery after TBI by inhibiting autophagy and neuroinflammation (Feng et al., 2017). Further experiments confirmed that TAK-242 mainly inhibited the TLR4-MyD88/TRIF-NF-κB signaling pathway to exert anti-neuroinflammatory activity. Ischemia/reperfusion (I/R) injury is a mechanism of brain injury after TBI (Hua et al., 2015). TAK-242 mitigates I/R injury by inhibiting the TLR-4 pathway, which is associated with microglial polarization to the M1 phenotype (Bell et al., 2013). The effects of TAK-242 on patients with TBI have a wide therapeutic time window which is from 4 hours to 5 days after injury (Zhang et al., 2014). However, all experimental results are from animal experiments; therefore, more clinical trials are needed to validate these findings.

### Erythropoietin

Erythropoietin is a well-known plasma factor that stimulates erythrocyte production and was first purified in 1977 (Witts, 1961; Miyake et al., 1977). EPO is mainly produced by interstitial cells in the adult kidney in response to hypoxia (Miyake et al., 1977; Kobayashi et al., 2017). In the late 1980s, recombinant human EPO (rh-EPO) became available for clinical use, revolutionizing the management of renal anemia. Now, more and more functions of EPO have been discovered, especially in neuroprotection (Hemani et al., 2021).

Erythropoietin significantly reduced brain tissue loss volume, ameliorated white matter injury, and improved neurobehavioral outcomes after ischemic stroke (Wang et al., 2017). The authors also demonstrated a shift from the M1 phenotype to the M2 phenotype at the infarct border after EPO treatment could attenuate brain injury. In a randomized, prospective clinical trial, it was confirmed that repeated, low-dose, rh-EPO treatment reduced the risk of disability for infants with moderate hypoxic-ischemic encephalopathy (HIE) without apparent side effects (Zhu et al., 2009).

The EPO receptor was shown to be highly expressed on the surface of microglia after brain injury, suggesting its role in the brain after an injury (Spandou et al., 2004). Reactive microglia are particularly effective at producing and releasing ROS/RNS (Block et al., 2007). Treatment with EPO activates the Akt/mTOR/NF-κB pathway, which is implicated in shifting macrophage activation state polarization from M1 to M2 (Xu et al., 2013). There is also evidence that EPO modulates neuroinflammation by decreasing levels of ROS/RNS, limiting microglial infiltration by preserving the health of the microvascular endothelial cells at the BBB (Bond and Rex, 2014). However, current clinical trial studies focus too much on the effectiveness and safety of EPO, but there is no corresponding clinical trial evidence on the method and time of administration.

### Colony-stimulating factor 1

CSF-1 (Colony-stimulating factor 1, namely Macrophage colony-stimulating factor) is the primary growth factor required for the control of monocyte and macrophage differentiation (Sehgal et al., 2021). The CSF-1 was initially purified from human urine in Stanley et al. (1975). Similar to rh-EPO, rh-CSF-1 was synthesized from the c-DNA of CSF-1 in the 1980s. CSF-1 has not been found substantial clinical application, but CSF-1 administration promotes microglia infiltration, differentiation, clearance of damaged cells and repair (Stanley et al., 1978). Therefore now scientists are interested in its use in neurological diseases.

The specific role of CSF-1 in microglia polarization is unclear, but it is known that CSF-1 significantly promotes proliferation and differentiation of microglia (M1, M2, or other types) (Stanley and Chitu, 2014). CSF-1 usually interacts with other molecules on microglia to enhance its role in controlling the direction of microglia polarization. This effect is similar to that of glucocorticoids that enhance the vasoconstriction of catecholamines by permissive action. CSF-1 upregulates TLR-4 and CD14 expression in microglia through ERK1/2 and p38, and thus promotes the LPS-induced microglia polarizing to proinflammatory phenotype (Parajuli et al., 2012). When CSF-1 is inhibited, this “permissiveness” effect is mitigated, reducing the inflammatory response. The inhibition of colony-stimulating factor 1 receptor (CSF-1R) exerted neuroprotection in ischemia cerebral stroke mice model through inhibiting microglia M1 polarization and NLRP3 pathway and increasing the balance function of injured mice (Liu et al., 2020).

Colony-stimulating factor 1 also plays an important role in TBI. An experiment by Li et al. (2020) showed that both immediate administration (within 24 h after injury) and the sequelae stage (3 months after injury) could effectively improve the recovery of cognitive function in m-TBI mice (Rajabi et al., 2021). Further studies showed that microglia activity was inhibited after drug administration, but the changes in microglia function were not elaborated. A previously published paper showed that eliminating chronically activated microglia by inhibitors of the CSF-1R could improve long-term neurological function after TBI (Henry et al., 2020). This experiment further indicated that if the inflammatory response of microglia after TBI could be precisely controlled, the therapeutic window of TBI will be greatly increased. As a cytokine targeting microglia, CSF-1 needs more basic and clinical studies on how to better apply it in clinical practice.

## Discussion

Traumatic brain injury has become a major health and socioeconomic problem worldwide. Primary injuries in TBI are largely irreversible, so the secondary damage stage becomes the only way to administer therapeutic measures. Neuroinflammation is the most important mechanism in secondary injury, and microglia play an important role in neuroinflammation. After microglial polarization, the cell phenotype changes, and its functions also change significantly. In this review, we have summarized that polarization of microglia to the M1 phenotype contributes to secondary damage after TBI, and M2 phenotype microglia aid in recovery from TBI. How to control the directions of polarization of microglia after TBI is an important consideration in the treatment of patients with TBI. We further summarized the cell-signaling pathways that were involved in microglial polarization after TBI, which provides a theoretical basis for further research and development of drugs targeting microglial polarization. In this paper, we also summarized some newly developed drugs and some new usages of old drugs that mainly inhibit the polarization of M1 microglia in the treatment of clinical patients. However, it is well known that M1 microglia are beneficial for a short period of time after TBI. Therefore, how to use existing clinical measures to detect when to use drugs to inhibit M1 microglia has become a current hotly discussed and difficult consideration, and more experiments are needed to put forward feasible measures in this aspect.

## Author contributions

Y-FL: literature collection and manuscript writing. XR: literature collection and manuscript writing. LZ: literature collection and manuscript writing. TC: validation, writing – review and editing, and supervision. Y-HW: Supervision. All authors contributed to the article and approved the submitted version.
